# Crystal structure of 4,4′-[(1,3,5,7-tetra­oxo-1,3,3a,4,4a,5,7,7a,8,8a-deca­hydro-4,8-etheno­pyrrolo­[3,4-*f*]iso­indole-2,6-di­yl)bis­(methyl­ene)]bis­(pyridin-1-ium) dinitrate

**DOI:** 10.1107/S2056989015022227

**Published:** 2015-11-25

**Authors:** Zhimin Liu

**Affiliations:** a School of Chemistry and Chemical Engineering, Shanxi University, Taiyuan 030006, People’s Republic of China

**Keywords:** crystal structure, salt, iso­indole, pyrrolo, pyridinium, nitrate(V) salt, N—H⋯O hydrogen bonds

## Abstract

In the title salt, C_24_H_22_N_4_O_4_^2+^·2NO_3_^−^, the cation is U-shaped with the two iso­indole dione rings inclined to one another by 60.41 (13)°, while the two outer pyridine rings are inclined to one another by 2.77 (12)°. The dihedral angles between the pyridine ring and the adjacent iso­indole dione ring are 71.82 (12) and 86.44 (13)°. In the crystal, each nitrate anion is linked to a protonated pyridine ring by N—H⋯O hydrogen bonds. These units are linked by a series of C—H⋯O hydrogen bonds, forming a three-dimensional structure.

## Related literature   

For the crystal structures of compounds with similar ligands, see: Yu *et al.* (2012 ▸); Li *et al.* (2011 ▸, 2012*a* ▸,*b* ▸). For the synthetic method used to prepare 2,6-bis­(pyridin-4-ylmeth­yl)-3a,4,4a,7a,8,8a-hexa­hydro-4,8-etheno­pyrrolo­[3,4-*f*]iso­indole-1,3,5,7(2*H*,6*H*)-tetra­one, see: Liu *et al.* (2007 ▸).
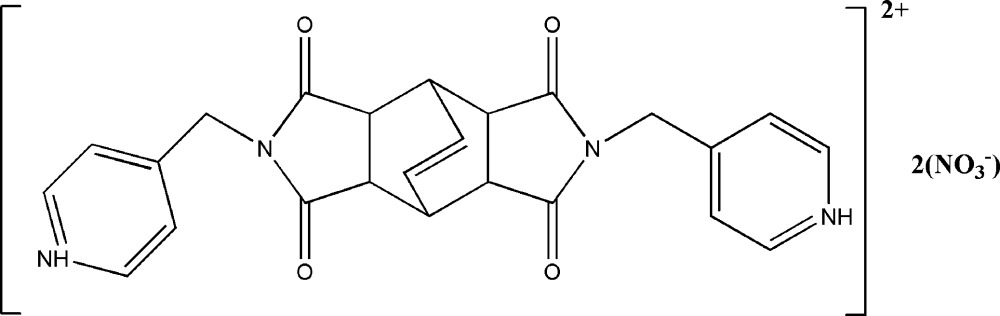


## Experimental   

### Crystal data  

C_24_H_22_N_4_O_4_^2+^·2NO_3_^−^
*M* *_r_* = 554.48
Monoclinic, 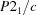

*a* = 13.0706 (6) Å
*b* = 14.3587 (5) Å
*c* = 12.9893 (5) Å
β = 104.861 (4)°
*V* = 2356.25 (16) Å^3^
*Z* = 4
Mo *K*α radiation
μ = 0.12 mm^−1^
*T* = 153 K
0.30 × 0.25 × 0.20 mm

### Data collection  

Bruker MWPC diffractometer
Absorption correction: multi-scan (*SADABS*; Bruker, 2004 ▸) *T*_min_ = 0.963, *T*_max_ = 0.976
13323 measured reflections
4555 independent reflections
2623 reflections with *I* > 2σ(*I*)
*R*_int_ = 0.031

### Refinement  

*R*[*F* ^2^ > 2σ(*F* ^2^)] = 0.043
*wR*(*F* ^2^) = 0.146
*S* = 1.00
4555 reflections
361 parameters
8 restraints
H-atom parameters constrained
Δρ_max_ = 0.23 e Å^−3^
Δρ_min_ = −0.28 e Å^−3^

### 

Data collection: *FRAMBO* (Bruker, 2004 ▸); cell refinement: *FRAMBO* and *SAINT* (Bruker, 2004 ▸); data reduction: *SAINT* (Bruker, 2004 ▸); program(s) used to solve structure: *SHELXS97* (Sheldrick, 2008 ▸); program(s) used to refine structure: *SHELXL97* (Sheldrick, 2008 ▸); molecular graphics: *SHELXTL* (Sheldrick, 2008 ▸); software used to prepare material for publication: *SHELXTL*.

## Supplementary Material

Crystal structure: contains datablock(s) I, New_Global_Publ_Block. DOI: 10.1107/S2056989015022227/su5240sup1.cif

Structure factors: contains datablock(s) I. DOI: 10.1107/S2056989015022227/su5240Isup2.hkl

Supporting information file. DOI: 10.1107/S2056989015022227/su5240Isup3.cml

**Fig. 1.**
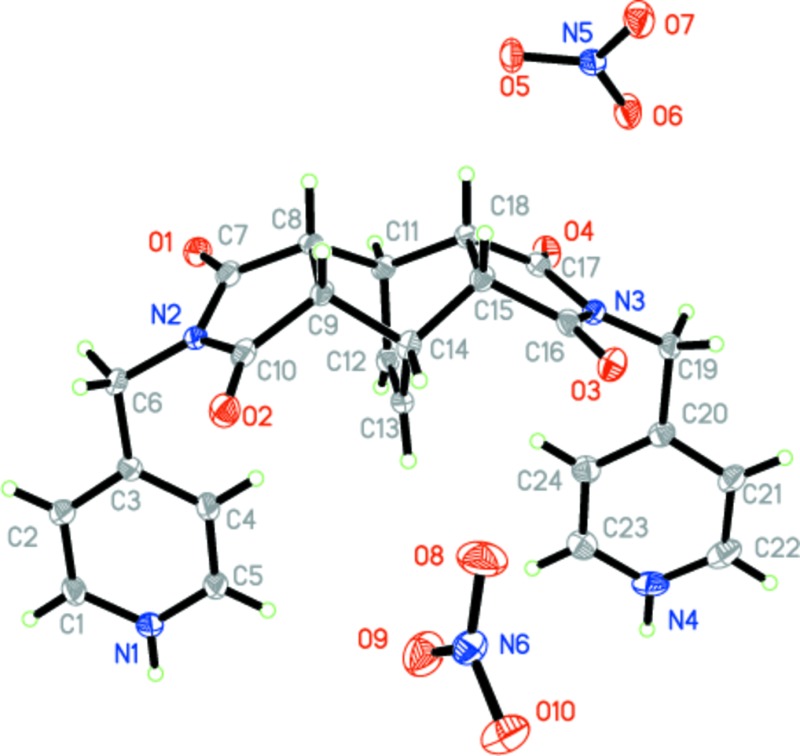
The molecular structure of the title salt, with atom labelling. Displacement ellipsoids are drawn at the 30% probability level.

**Fig. 2.**
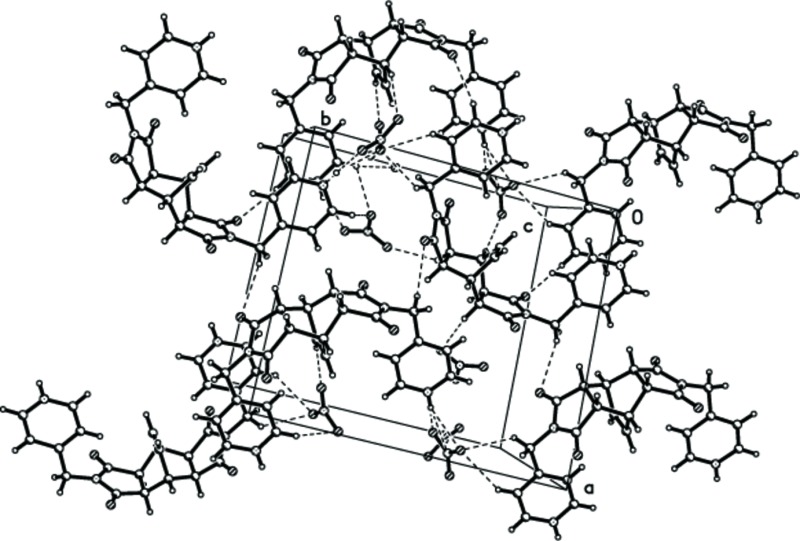
Crystal packing of the title salt, viewed along the c axis. hydrogen bonds are shown as dashed lines (see Table 1).

. DOI: 10.1107/S2056989015022227/su5240fig1.tif
The mol­ecular structure of the title salt, with atom labelling. Displacement ellipsoids are drawn at the 30% probability level.

c . DOI: 10.1107/S2056989015022227/su5240fig2.tif
Crystal packing of the title salt, viewed along the *c* axis. hydrogen bonds are shown as dashed lines (see Table 1).

CCDC reference: 1434635

Additional supporting information:  crystallographic information; 3D view; checkCIF report

## Figures and Tables

**Table 1:** Hydrogen-bond geometry (Å, °)

*D*—H⋯*A*	*D*—H	H⋯*A*	*D*⋯*A*	*D*—H⋯*A*
N1—H1*A*⋯O6^i^	0.86	2.40	3.096 (3)	138
N1—H1*A*⋯O7^i^	0.86	1.90	2.728 (3)	161
N4—H4*A*⋯O9^ii^	0.86	1.93	2.771 (3)	164
C6—H6*B*⋯O3^iii^	0.97	2.57	3.386 (3)	142
C8—H8*A*⋯O8	0.98	2.30	3.155 (3)	145
C11—H11*A*⋯O5^iv^	0.98	2.48	3.386 (3)	153
C13—H13*A*⋯O5^v^	0.93	2.30	3.121 (3)	147
C14—H14*A*⋯O2^iv^	0.98	2.54	3.403 (3)	147
C19—H19*A*⋯O6^vi^	0.97	2.55	3.246 (3)	129
C21—H21*A*⋯O6^vi^	0.93	2.56	3.362 (3)	145
C22—H22*A*⋯O2^vii^	0.93	2.43	3.257 (3)	149
C23—H23*A*⋯O7^v^	0.93	2.56	3.345 (3)	143

Symmetry codes: (i) 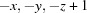; (ii) 
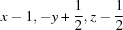
; (iii) 
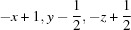
; (iv) 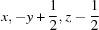; (v) 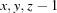; (vi) 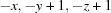; (vii) 
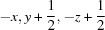
.

## supplementary crystallographic information

### S1. Comment

In recent years, complexes with terminal pyridyl-substituted ligands have been used to construct various metal-organic frameworks (MOFs). MOFs have aroused considerable inter­ests not only owing to their novel topological structures and for their potential applications (Li *et al.*, 2011, 2012a,b; Yu *et al.*, 2012). The title salt was synthesized from the reaction of

2,6-bis­(pyridin-4-yl­methyl)-3*a*,4,4*a*,7*a*,8,8*a*-hexa­hydro-4,8-etheno­pyrrolo­[3,4-*f*]iso­indole-1,3,5,7(2*H*,6*H*)-tetra­one and nitric acid in chloro­form.

In the title salt, Fig. 1, the cation has two terminal protonated pyridine N atoms, N1 and N4. The backbone of the cation has essentially a *cis*-U conformation. The two iso­indole dione rings (N2/C7—C10 and N3/C15—C18) are inclined to one another by 60.41 (13) °, while the two outer pyridine rings (N1/C1—C5 and N4/C20—C24) are inclined to one another by 2.77 (12) °. The dihedral angles between the pyridine ring and the adjacent iso­indole dione ring are 86.44 (13) ° for rings N1/C1—C5 and N2/C7—C10, and 71.82 (12)° for rings N4/C20—C24 and N3/C15—C18. The bond angles C3—C6—N2 and C20—C19—N3 are 114.1 (2) and 113.52 (19) °, respectively, larger than normal (109 °)

In the crystal, each anion is linked to a protonated pyridine ring by N—H···O hydrogen bonds (Table 1). These units are linked by a series of C—H···O hydrogen bonds forming a three-dimensional structure (Table 1 and Fig. 2).

### S2. Synthesis and crystallization

The compound 2,6-bis­(pyridin-4-yl­methyl)-3*a*,4,4*a*,7*a*,8,8*a*-hexa­hydro-4,8-etheno­pyrrolo­[3,4-*f*]iso­indole-1,3,5,7(2*H*,6*H*)-tetra­one (L) was prepared according to a reported procedure (Liu *et al.*, 2007). L (1 mmol, 0.43g) was added to CHCl_3_ (5 ml) under vigorous stirring. The clear solution was combined with nitric(V) acid (0.1 M, 1 ml) and stirred for 20 min. The resulting solution was left to crystallize at ambient temperature. After two weeks, large block-shaped yellow single crystals of the title salt suitable for X-ray diffraction analysis were obtained [yield: 73%; based on the 4-(amino­methyl)-pyridine].

### S3. Refinement details

Crystal data, data collection and structure refinement details are summarized in Table 2. The H atoms were placed in calculated positions and refined in a riding-model approximation: N—H = 0.86 Å and C—H = 0.93-0.98 Å with U_iso_(H) = 1.2U_eq_(N,C).

### Crystal data

**Table:**

C_24_H_22_N_4_O_4_^2+^·2NO_3_^−^	*F*(000) = 1152
*M**_r_* = 554.48	*D*_x_ = 1.563 Mg m^−^^3^
Monoclinic, *P*2_1_/*c*	Mo *K*α radiation, λ = 0.71073 Å
Hall symbol: -P 2ybc	Cell parameters from 4348 reflections
*a* = 13.0706 (6) Å	θ = 2.8–29.7°
*b* = 14.3587 (5) Å	µ = 0.12 mm^−^^1^
*c* = 12.9893 (5) Å	*T* = 153 K
β = 104.861 (4)°	Block, yellow
*V* = 2356.25 (16) Å^3^	0.30 × 0.25 × 0.20 mm
*Z* = 4	

### Data collection

**Table:**

Bruker MWPC diffractometer	4555 independent reflections
Radiation source: fine-focus sealed tube	2623 reflections with *I* > 2σ(*I*)
Graphite monochromator	*R*_int_ = 0.031
Detector resolution: 16.08 pixels mm^-1^	θ_max_ = 26.0°, θ_min_ = 2.8°
phi and ω scans	*h* = −16→12
Absorption correction: multi-scan (*SADABS*; Bruker, 2004)	*k* = −17→17
*T*_min_ = 0.963, *T*_max_ = 0.976	*l* = −15→16
13323 measured reflections	

### Refinement

**Table:**

Refinement on *F*^2^	Primary atom site location: structure-invariant direct methods
Least-squares matrix: full	Secondary atom site location: difference Fourier map
*R*[*F*^2^ > 2σ(*F*^2^)] = 0.043	Hydrogen site location: inferred from neighbouring sites
*wR*(*F*^2^) = 0.146	H-atom parameters constrained
*S* = 1.00	*w* = 1/[σ^2^(*F*_o_^2^) + (0.0861*P*)^2^] where *P* = (*F*_o_^2^ + 2*F*_c_^2^)/3
4555 reflections	(Δ/σ)_max_ < 0.001
361 parameters	Δρ_max_ = 0.23 e Å^−^^3^
8 restraints	Δρ_min_ = −0.28 e Å^−^^3^

### Special details

**Table:**

Geometry. All e.s.d.'s (except the e.s.d. in the dihedral angle between two l.s. planes) are estimated using the full covariance matrix. The cell e.s.d.'s are taken into account individually in the estimation of e.s.d.'s in distances, angles and torsion angles; correlations between e.s.d.'s in cell parameters are only used when they are defined by crystal symmetry. An approximate (isotropic) treatment of cell e.s.d.'s is used for estimating e.s.d.'s involving l.s. planes.
Refinement. Refinement of *F*^2^ against ALL reflections. The weighted *R*-factor *wR* and goodness of fit *S* are based on *F*^2^, conventional *R*-factors *R* are based on *F*, with *F* set to zero for negative *F*^2^. The threshold expression of *F*^2^ > σ(*F*^2^) is used only for calculating *R*-factors(gt) *etc*. and is not relevant to the choice of reflections for refinement. *R*-factors based on *F*^2^ are statistically about twice as large as those based on *F*, and *R*- factors based on ALL data will be even larger.

### Fractional atomic coordinates and isotropic or equivalent isotropic displacement parameters (Å^2^)

**Table:**

	*x*	*y*	*z*	*U*_iso_*/*U*_eq_	
O1	0.50387 (14)	0.20676 (12)	0.21910 (13)	0.0393 (5)	
O3	0.28425 (13)	0.55976 (12)	0.17390 (13)	0.0359 (4)	
O4	0.11752 (14)	0.46314 (12)	0.42145 (14)	0.0405 (5)	
N6	0.70026 (17)	0.29400 (14)	0.54704 (17)	0.0345 (5)	
O2	0.38605 (14)	0.12774 (12)	0.50720 (13)	0.0383 (4)	
N3	0.18131 (15)	0.52033 (12)	0.28596 (15)	0.0274 (5)	
N4	−0.16669 (16)	0.40594 (14)	0.05847 (16)	0.0330 (5)	
H4A	−0.2196	0.3725	0.0256	0.040*	
O10	0.75841 (16)	0.28012 (13)	0.63718 (15)	0.0479 (5)	
C7	0.47472 (18)	0.21943 (16)	0.29881 (19)	0.0288 (5)	
N2	0.44842 (15)	0.14774 (12)	0.36012 (15)	0.0273 (5)	
O9	0.68040 (16)	0.22794 (12)	0.48063 (15)	0.0507 (5)	
C20	−0.00046 (18)	0.51242 (16)	0.16821 (19)	0.0281 (5)	
C14	0.37646 (18)	0.37073 (15)	0.26654 (17)	0.0256 (5)	
H14A	0.4047	0.3876	0.2061	0.031*	
C15	0.35286 (18)	0.45870 (16)	0.32628 (17)	0.0265 (5)	
H15A	0.4182	0.4932	0.3573	0.032*	
O8	0.66218 (15)	0.37204 (12)	0.52061 (15)	0.0505 (5)	
C3	0.36003 (19)	0.00449 (16)	0.27263 (18)	0.0292 (6)	
C23	−0.0807 (2)	0.36424 (18)	0.1159 (2)	0.0365 (6)	
H23A	−0.0773	0.2996	0.1185	0.044*	
C10	0.41200 (19)	0.17942 (17)	0.44436 (18)	0.0282 (5)	
C9	0.40865 (18)	0.28479 (15)	0.44104 (18)	0.0264 (5)	
H9A	0.4514	0.3109	0.5080	0.032*	
C4	0.2756 (2)	0.05567 (19)	0.21382 (19)	0.0345 (6)	
H4B	0.2780	0.1204	0.2161	0.041*	
C6	0.46021 (19)	0.04991 (15)	0.3381 (2)	0.0307 (6)	
H6A	0.4835	0.0169	0.4052	0.037*	
H6B	0.5151	0.0435	0.3005	0.037*	
C11	0.2933 (2)	0.32076 (15)	0.41775 (19)	0.0297 (6)	
H11A	0.2586	0.3000	0.4723	0.036*	
C18	0.29907 (18)	0.42838 (14)	0.41363 (17)	0.0246 (5)	
H18A	0.3379	0.4531	0.4830	0.030*	
C2	0.3535 (2)	−0.09187 (16)	0.2683 (2)	0.0348 (6)	
H2A	0.4078	−0.1281	0.3092	0.042*	
C13	0.27571 (19)	0.31613 (15)	0.23084 (19)	0.0295 (6)	
H13A	0.2449	0.3026	0.1596	0.035*	
C17	0.1902 (2)	0.47097 (15)	0.37992 (19)	0.0305 (6)	
C21	−0.09112 (18)	0.55362 (17)	0.10493 (19)	0.0326 (6)	
H21A	−0.0960	0.6181	0.0997	0.039*	
C8	0.45595 (18)	0.31102 (16)	0.34811 (18)	0.0262 (5)	
H8A	0.5228	0.3446	0.3744	0.031*	
N1	0.18676 (18)	−0.08144 (16)	0.14709 (18)	0.0438 (6)	
H1A	0.1327	−0.1085	0.1062	0.053*	
C16	0.27339 (19)	0.51993 (16)	0.2524 (2)	0.0295 (5)	
C12	0.23399 (18)	0.28860 (15)	0.30875 (19)	0.0305 (6)	
H12A	0.1731	0.2524	0.2968	0.037*	
C24	0.0034 (2)	0.41598 (17)	0.1715 (2)	0.0393 (7)	
H24A	0.0636	0.3862	0.2119	0.047*	
C5	0.1890 (2)	0.01172 (19)	0.1526 (2)	0.0392 (6)	
H5A	0.1315	0.0463	0.1146	0.047*	
C19	0.08681 (19)	0.57240 (16)	0.2337 (2)	0.0316 (6)	
H19A	0.1056	0.6191	0.1879	0.038*	
H19B	0.0606	0.6046	0.2875	0.038*	
C22	−0.1736 (2)	0.49923 (17)	0.0501 (2)	0.0365 (6)	
H22A	−0.2341	0.5268	0.0074	0.044*	
C1	0.2658 (2)	−0.1332 (2)	0.2030 (2)	0.0454 (7)	
H1B	0.2618	−0.1977	0.1979	0.055*	
N5	0.01934 (18)	0.21064 (14)	0.97829 (19)	0.0381 (5)	
O7	−0.03711 (16)	0.15681 (13)	1.01790 (16)	0.0491 (5)	
O6	−0.01019 (17)	0.23019 (13)	0.88230 (16)	0.0542 (6)	
O5	0.10141 (15)	0.24289 (15)	1.03608 (17)	0.0569 (6)	

### Atomic displacement parameters (Å^2^)

**Table:**

	*U*^11^	*U*^22^	*U*^33^	*U*^12^	*U*^13^	*U*^23^
O1	0.0449 (11)	0.0410 (10)	0.0353 (10)	0.0081 (9)	0.0161 (9)	0.0021 (8)
O3	0.0375 (10)	0.0340 (9)	0.0353 (10)	0.0000 (8)	0.0075 (8)	0.0069 (8)
O4	0.0428 (11)	0.0357 (10)	0.0486 (11)	0.0037 (9)	0.0220 (9)	−0.0010 (9)
N6	0.0299 (12)	0.0288 (11)	0.0384 (13)	−0.0005 (10)	−0.0030 (10)	−0.0065 (10)
O2	0.0471 (11)	0.0318 (9)	0.0365 (10)	0.0004 (9)	0.0115 (9)	0.0056 (8)
N3	0.0234 (11)	0.0213 (10)	0.0335 (11)	−0.0013 (8)	0.0002 (8)	0.0004 (9)
N4	0.0269 (11)	0.0307 (11)	0.0383 (12)	−0.0027 (9)	0.0028 (10)	−0.0045 (10)
O10	0.0567 (13)	0.0399 (11)	0.0374 (11)	0.0075 (10)	−0.0058 (10)	−0.0023 (9)
C7	0.0241 (13)	0.0294 (13)	0.0296 (13)	0.0005 (11)	0.0009 (11)	0.0015 (11)
N2	0.0266 (11)	0.0220 (10)	0.0308 (11)	−0.0004 (8)	0.0025 (9)	−0.0005 (8)
O9	0.0549 (13)	0.0311 (10)	0.0512 (12)	0.0098 (9)	−0.0139 (10)	−0.0130 (9)
C20	0.0240 (13)	0.0282 (13)	0.0323 (13)	0.0001 (10)	0.0073 (10)	−0.0018 (11)
C14	0.0291 (13)	0.0238 (11)	0.0235 (12)	−0.0006 (10)	0.0059 (10)	−0.0016 (10)
C15	0.0224 (12)	0.0247 (11)	0.0279 (12)	−0.0040 (9)	−0.0014 (9)	0.0011 (10)
O8	0.0499 (12)	0.0259 (10)	0.0589 (13)	0.0104 (9)	−0.0169 (10)	−0.0041 (9)
C3	0.0328 (14)	0.0267 (12)	0.0293 (13)	−0.0033 (11)	0.0101 (11)	−0.0057 (11)
C23	0.0309 (15)	0.0273 (13)	0.0459 (16)	0.0012 (11)	−0.0001 (12)	−0.0039 (12)
C10	0.0266 (13)	0.0311 (13)	0.0245 (13)	0.0029 (11)	0.0022 (11)	0.0040 (11)
C9	0.0302 (14)	0.0238 (12)	0.0242 (12)	−0.0007 (10)	0.0051 (10)	−0.0001 (10)
C4	0.0341 (15)	0.0364 (14)	0.0313 (14)	−0.0014 (12)	0.0051 (12)	−0.0025 (11)
C6	0.0338 (15)	0.0209 (12)	0.0356 (14)	0.0036 (11)	0.0055 (11)	−0.0013 (11)
C11	0.0367 (15)	0.0221 (11)	0.0314 (14)	0.0030 (11)	0.0108 (11)	0.0025 (10)
C18	0.0288 (13)	0.0192 (11)	0.0220 (12)	−0.0012 (10)	−0.0005 (10)	−0.0017 (9)
C2	0.0405 (16)	0.0255 (13)	0.0420 (15)	−0.0034 (12)	0.0171 (13)	−0.0030 (12)
C13	0.0311 (14)	0.0234 (12)	0.0295 (13)	0.0035 (10)	−0.0004 (11)	−0.0051 (10)
C17	0.0372 (15)	0.0212 (12)	0.0322 (13)	−0.0012 (11)	0.0074 (11)	−0.0024 (10)
C21	0.0309 (15)	0.0276 (13)	0.0366 (14)	−0.0014 (11)	0.0038 (12)	−0.0008 (11)
C8	0.0249 (13)	0.0258 (12)	0.0261 (12)	−0.0008 (10)	0.0034 (10)	0.0027 (10)
N1	0.0359 (13)	0.0504 (15)	0.0446 (14)	−0.0157 (11)	0.0093 (11)	−0.0168 (11)
C16	0.0328 (14)	0.0235 (12)	0.0317 (14)	−0.0019 (11)	0.0073 (11)	−0.0012 (11)
C12	0.0221 (13)	0.0216 (12)	0.0451 (15)	0.0014 (10)	0.0037 (11)	−0.0016 (11)
C24	0.0296 (15)	0.0277 (14)	0.0526 (17)	0.0059 (11)	−0.0038 (12)	−0.0004 (12)
C5	0.0379 (16)	0.0446 (16)	0.0337 (15)	−0.0001 (13)	0.0068 (12)	−0.0047 (12)
C19	0.0317 (14)	0.0198 (12)	0.0406 (14)	0.0018 (10)	0.0042 (11)	0.0007 (10)
C22	0.0303 (15)	0.0339 (14)	0.0400 (15)	0.0069 (12)	−0.0005 (12)	0.0008 (12)
C1	0.0553 (19)	0.0345 (15)	0.0539 (18)	−0.0135 (14)	0.0276 (15)	−0.0112 (13)
N5	0.0361 (13)	0.0255 (11)	0.0519 (15)	−0.0037 (10)	0.0099 (11)	−0.0078 (11)
O7	0.0555 (13)	0.0414 (11)	0.0542 (12)	−0.0158 (10)	0.0212 (10)	−0.0075 (9)
O6	0.0622 (14)	0.0435 (12)	0.0481 (13)	−0.0046 (10)	−0.0016 (11)	0.0059 (10)
O5	0.0337 (12)	0.0590 (13)	0.0703 (14)	−0.0121 (10)	−0.0004 (10)	−0.0196 (11)

### Geometric parameters (Å, º)

**Table:**

O1—C7	1.205 (3)	C9—C8	1.537 (3)
O3—C16	1.209 (3)	C9—C11	1.549 (3)
O4—C17	1.212 (3)	C9—H9A	0.9800
N6—O10	1.238 (3)	C4—C5	1.360 (3)
N6—O8	1.238 (3)	C4—H4B	0.9300
N6—O9	1.263 (2)	C6—H6A	0.9700
O2—C10	1.214 (3)	C6—H6B	0.9700
N3—C16	1.381 (3)	C11—C12	1.502 (3)
N3—C17	1.390 (3)	C11—C18	1.549 (3)
N3—C19	1.454 (3)	C11—H11A	0.9800
N4—C23	1.322 (3)	C18—C17	1.507 (3)
N4—C22	1.345 (3)	C18—H18A	0.9800
N4—H4A	0.8600	C2—C1	1.373 (4)
C7—N2	1.397 (3)	C2—H2A	0.9300
C7—C8	1.510 (3)	C13—C12	1.327 (3)
N2—C10	1.378 (3)	C13—H13A	0.9300
N2—C6	1.450 (3)	C21—C22	1.373 (3)
C20—C24	1.386 (3)	C21—H21A	0.9300
C20—C21	1.389 (3)	C8—H8A	0.9800
C20—C19	1.506 (3)	N1—C1	1.328 (4)
C14—C13	1.500 (3)	N1—C5	1.339 (3)
C14—C8	1.540 (3)	N1—H1A	0.8600
C14—C15	1.554 (3)	C12—H12A	0.9300
C14—H14A	0.9800	C24—H24A	0.9300
C15—C16	1.504 (3)	C5—H5A	0.9300
C15—C18	1.543 (3)	C19—H19A	0.9700
C15—H15A	0.9800	C19—H19B	0.9700
C3—C4	1.382 (3)	C22—H22A	0.9300
C3—C2	1.387 (3)	C1—H1B	0.9300
C3—C6	1.514 (3)	N5—O5	1.231 (3)
C23—C24	1.369 (3)	N5—O6	1.239 (3)
C23—H23A	0.9300	N5—O7	1.265 (3)
C10—C9	1.514 (3)		
			
O10—N6—O8	120.9 (2)	C12—C11—H11A	111.4
O10—N6—O9	119.6 (2)	C9—C11—H11A	111.4
O8—N6—O9	119.5 (2)	C18—C11—H11A	111.4
C16—N3—C17	113.1 (2)	C17—C18—C15	104.26 (18)
C16—N3—C19	124.03 (19)	C17—C18—C11	111.34 (19)
C17—N3—C19	122.77 (19)	C15—C18—C11	110.03 (18)
C23—N4—C22	121.7 (2)	C17—C18—H18A	110.4
C23—N4—H4A	119.1	C15—C18—H18A	110.4
C22—N4—H4A	119.1	C11—C18—H18A	110.4
O1—C7—N2	123.8 (2)	C1—C2—C3	119.3 (3)
O1—C7—C8	128.1 (2)	C1—C2—H2A	120.4
N2—C7—C8	108.02 (18)	C3—C2—H2A	120.4
C10—N2—C7	113.26 (19)	C12—C13—C14	114.8 (2)
C10—N2—C6	123.56 (19)	C12—C13—H13A	122.6
C7—N2—C6	123.17 (19)	C14—C13—H13A	122.6
C24—C20—C21	117.6 (2)	O4—C17—N3	122.7 (2)
C24—C20—C19	122.5 (2)	O4—C17—C18	128.5 (2)
C21—C20—C19	119.9 (2)	N3—C17—C18	108.77 (19)
C13—C14—C8	107.79 (18)	C22—C21—C20	120.1 (2)
C13—C14—C15	107.95 (18)	C22—C21—H21A	119.9
C8—C14—C15	107.11 (17)	C20—C21—H21A	119.9
C13—C14—H14A	111.3	C7—C8—C9	105.16 (18)
C8—C14—H14A	111.3	C7—C8—C14	110.32 (19)
C15—C14—H14A	111.3	C9—C8—C14	109.90 (18)
C16—C15—C18	105.29 (18)	C7—C8—H8A	110.4
C16—C15—C14	110.47 (18)	C9—C8—H8A	110.4
C18—C15—C14	108.91 (17)	C14—C8—H8A	110.4
C16—C15—H15A	110.7	C1—N1—C5	121.7 (2)
C18—C15—H15A	110.7	C1—N1—H1A	119.1
C14—C15—H15A	110.7	C5—N1—H1A	119.1
C4—C3—C2	118.4 (2)	O3—C16—N3	124.4 (2)
C4—C3—C6	122.3 (2)	O3—C16—C15	127.1 (2)
C2—C3—C6	119.2 (2)	N3—C16—C15	108.45 (19)
N4—C23—C24	120.2 (2)	C13—C12—C11	114.4 (2)
N4—C23—H23A	119.9	C13—C12—H12A	122.8
C24—C23—H23A	119.9	C11—C12—H12A	122.8
O2—C10—N2	123.0 (2)	C23—C24—C20	120.5 (2)
O2—C10—C9	128.2 (2)	C23—C24—H24A	119.7
N2—C10—C9	108.71 (19)	C20—C24—H24A	119.7
C10—C9—C8	104.63 (18)	N1—C5—C4	119.9 (3)
C10—C9—C11	111.01 (19)	N1—C5—H5A	120.1
C8—C9—C11	109.41 (18)	C4—C5—H5A	120.1
C10—C9—H9A	110.5	N3—C19—C20	113.52 (19)
C8—C9—H9A	110.5	N3—C19—H19A	108.9
C11—C9—H9A	110.5	C20—C19—H19A	108.9
C5—C4—C3	120.2 (2)	N3—C19—H19B	108.9
C5—C4—H4B	119.9	C20—C19—H19B	108.9
C3—C4—H4B	119.9	H19A—C19—H19B	107.7
N2—C6—C3	114.1 (2)	N4—C22—C21	119.8 (2)
N2—C6—H6A	108.7	N4—C22—H22A	120.1
C3—C6—H6A	108.7	C21—C22—H22A	120.1
N2—C6—H6B	108.7	N1—C1—C2	120.4 (3)
C3—C6—H6B	108.7	N1—C1—H1B	119.8
H6A—C6—H6B	107.6	C2—C1—H1B	119.8
C12—C11—C9	108.83 (18)	O5—N5—O6	121.7 (2)
C12—C11—C18	106.99 (19)	O5—N5—O7	119.3 (2)
C9—C11—C18	106.61 (19)	O6—N5—O7	118.9 (2)

### Hydrogen-bond geometry (Å, º)

**Table:**

*D*—H···*A*	*D*—H	H···*A*	*D*···*A*	*D*—H···*A*
N1—H1*A*···O6^i^	0.86	2.40	3.096 (3)	138
N1—H1*A*···O7^i^	0.86	1.90	2.728 (3)	161
N4—H4*A*···O9^ii^	0.86	1.93	2.771 (3)	164
C6—H6*B*···O3^iii^	0.97	2.57	3.386 (3)	142
C8—H8*A*···O8	0.98	2.30	3.155 (3)	145
C11—H11*A*···O5^iv^	0.98	2.48	3.386 (3)	153
C13—H13*A*···O5^v^	0.93	2.30	3.121 (3)	147
C14—H14*A*···O2^iv^	0.98	2.54	3.403 (3)	147
C19—H19*A*···O6^vi^	0.97	2.55	3.246 (3)	129
C21—H21*A*···O6^vi^	0.93	2.56	3.362 (3)	145
C22—H22*A*···O2^vii^	0.93	2.43	3.257 (3)	149
C23—H23*A*···O7^v^	0.93	2.56	3.345 (3)	143

Symmetry codes: (i) −*x*, −*y*, −*z*+1; (ii) *x*−1, −*y*+1/2, *z*−1/2; (iii) −*x*+1, *y*−1/2, −*z*+1/2; (iv) *x*, −*y*+1/2, *z*−1/2; (v) *x*, *y*, *z*−1; (vi) −*x*, −*y*+1, −*z*+1; (vii) −*x*, *y*+1/2, −*z*+1/2.
