# Supplementary figures and images for: Crystal structure of 4,4′-[(1,3,5,7-tetra­oxo-1,3,3a,4,4a,5,7,7a,8,8a-deca­hydro-4,8-etheno­pyrrolo­[3,4-f]iso­indole-2,6-di­yl)bis­(methyl­ene)]bis­(pyridin-1-ium) dinitrate
Source: Acta Crystallogr E Crystallogr Commun. 2015 Nov 25;71(Pt 12):o986–7. doi: 10.1107/S2056989015022227 (PMC4719937; doi:10.1107/S2056989015022227)

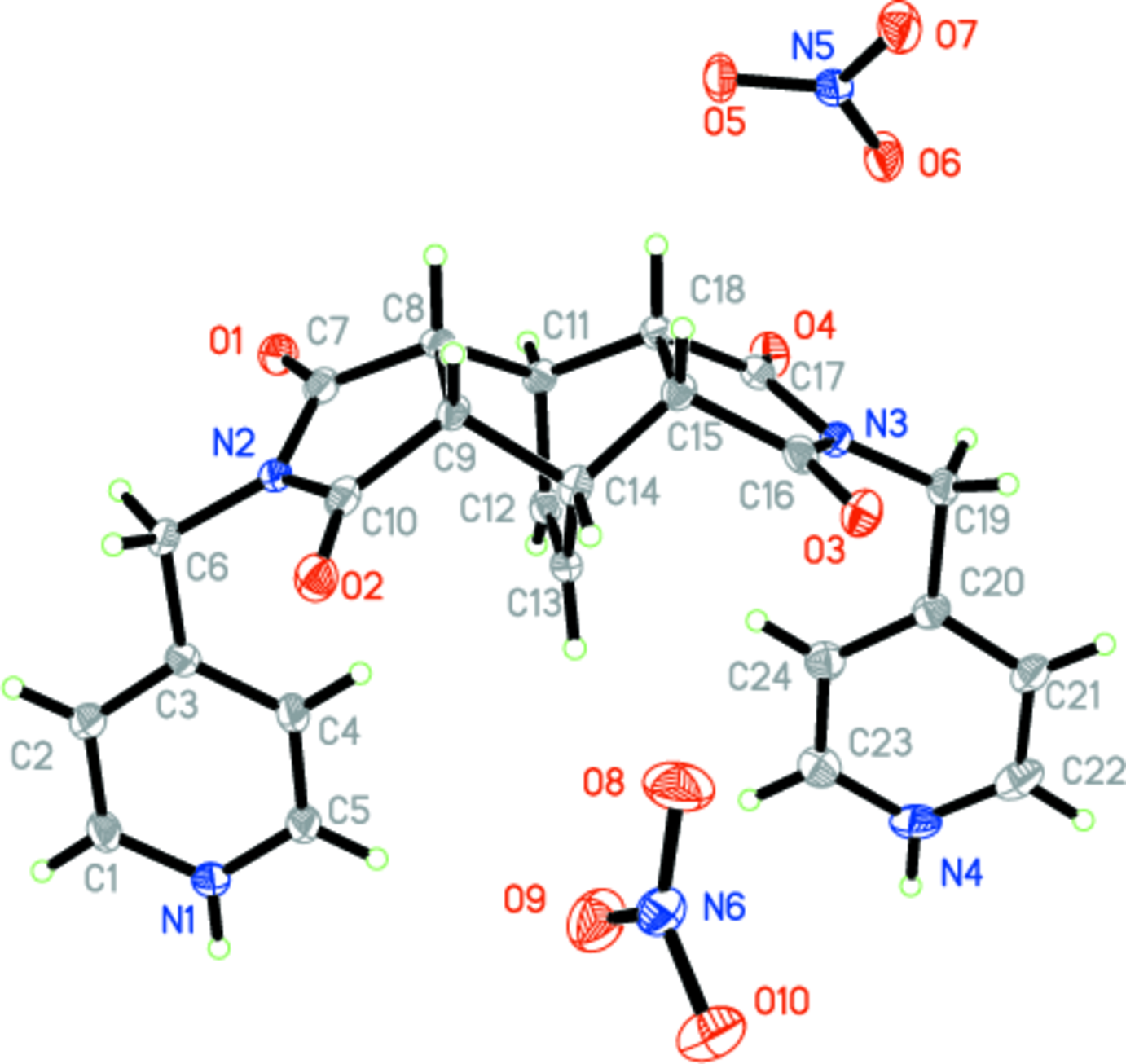

Supplement: Supplementary file 4 [file e-71-0o986-fig1.tif]

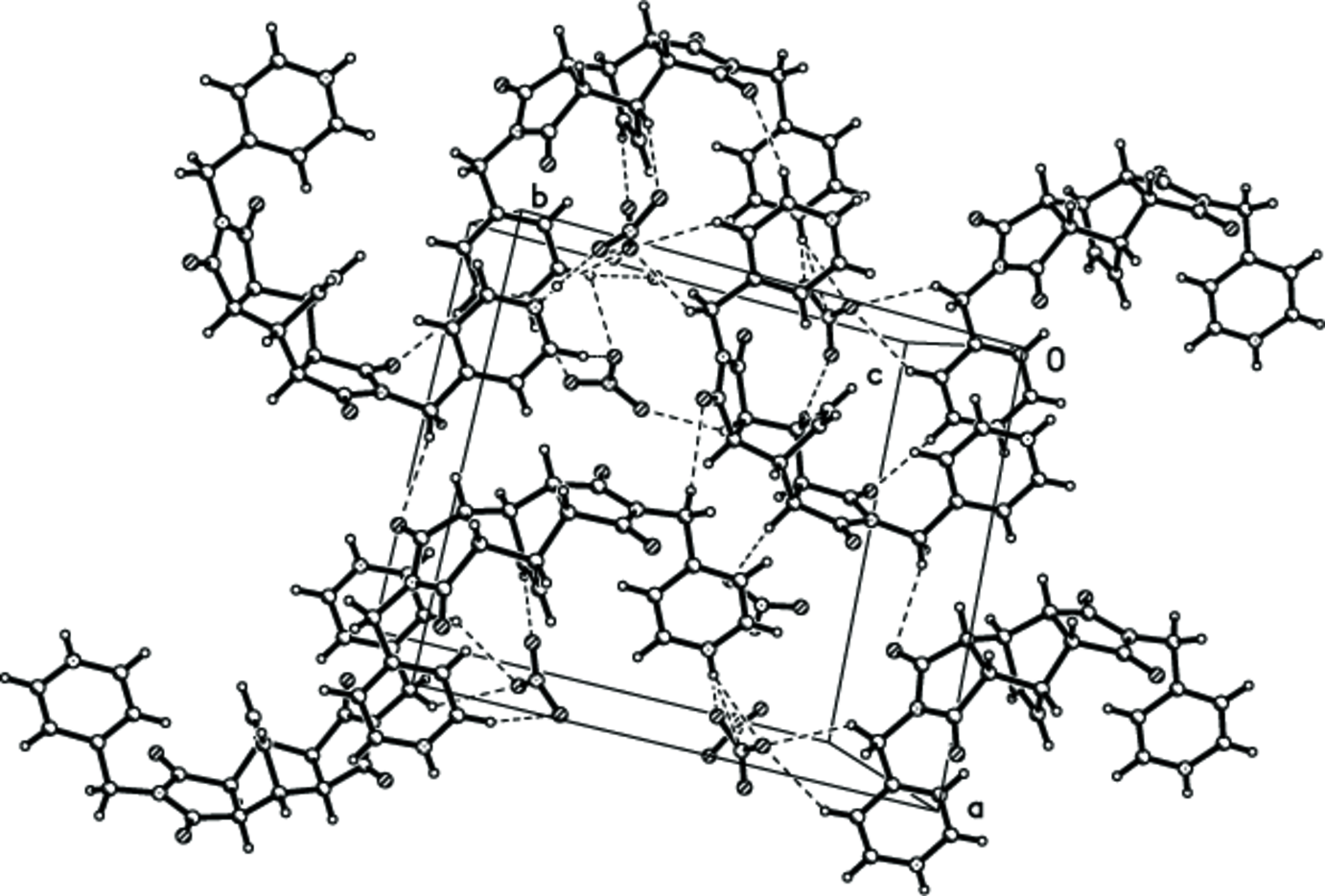

Supplement: Supplementary file 5 [file e-71-0o986-fig2.tif]
